# Anomalous self-experience, body image disturbance, and eating disorder symptomatology in first-onset anorexia nervosa

**DOI:** 10.1007/s40519-021-01145-0

**Published:** 2021-03-04

**Authors:** Lorenzo Moccia, Eliana Conte, Marianna Ambrosecchia, Delfina Janiri, Salvatore Di Pietro, Valentina De Martin, Marco Di Nicola, Lucio Rinaldi, Gabriele Sani, Vittorio Gallese, Luigi Janiri

**Affiliations:** 1 Section of Psychiatry, Department of Neuroscience, Università Cattolica del Sacro Cuore, Rome, Italy; 2 Department of Psychiatry, Fondazione Policlinico Universitario “A. Gemelli” IRCCS, L.go Agostino Gemelli 8, 00168 Rome, Italy; 3 Eating Disorders Treatment Unit, Casa di Cura Villa Armonia Nuova, Rome, Italy; 4 Unit of Neuroscience, Department of Medicine and Surgery, University of Parma, Parma, Italy; 5 Department of Psychiatry and Neurology, Sapienza University of Rome, Rome, Italy

**Keywords:** Self-disorder, Minimal self, Eating disorders, Anorexia nervosa, Restrictive type, Body image, Transdiagnostic feature, Hilde Bruch

## Abstract

**Purpose:**

Anorexia nervosa-restrictive subtype (AN-R) is a life-threatening disorder relying on behavioural abnormalities, such as excessive food restriction or exercise. Such abnormalities may be secondary to an “objectified” attitude toward body image and self. This is the first study exploring the impact of anomalous self-experience (ASEs) on abnormal body image attitude and eating disorder (ED) symptomatology in individuals with AN-R at onset.

**Methods:**

We recruited Italian female participants, 40 with AN-R (mean age 18.3 ± 2.3) and 45 age and educational level-matched healthy controls (HCs) (mean age 18.2 ± 2.6). ASEs, body image attitude, and ED symptom severity were assessed through the examination of anomalous self-experience (EASE), the body uneasiness test (BUT), and the eating disorder examination questionnaire (EDE-Q), respectively. We conducted multivariate analysis of variance to investigate distribution patterns of variables of interest, and mediation analysis to test the effect of ASEs and body image on ED symptomatology.

**Results:**

Individuals with AN-R scored higher than HCs on the EASE (*p* < .0001). A direct effect of ASEs on ED severity (*p* = 0.009; bootstrapped LLCI = 0.067, ULCI = 0.240) was found in AN-R. After modelling the effect of abnormal body image attitude, the relationship between EASE total score and ED symptomatology was significantly mediated by BUT (*p* = 0.002; bootstrapped LLCI = 0.001, ULCI = 0.172).

**Conclusion:**

Although the exact pathways linking AN-R to self-disorder remain to be identified, a broader exploration of transdiagnostic features in AN, including explorations of different dimensions of self-experience and intersubjectivity, may shed further light on the clinical phenomenology of the disorder.

**Level of evidence:**

Level III, case–control analytic study.

**Supplementary Information:**

The online version contains supplementary material available at 10.1007/s40519-021-01145-0.

## Introduction

Anorexia nervosa (AN) is a severe and often life-threatening disorder whose onset usually occurs during adolescence and early adulthood. AN may manifest in a restrictive subtype (AN-R) where severe weight loss and starvation may potentially lead to a multitude of adverse medical sequelae, including cardiovascular, metabolic, endocrine, and other disturbances that increase the susceptibility to disease, as well as to increased risk of premature mortality [1].

Anorexia nervosa-restrictive subtype is usually defined on the basis of behavioural abnormalities, such as excessive food restriction or exercise; however, there is a general agreement that these are epiphenomena secondary to a deeper psychopathological core, which may involve the way individuals with AN-R experience their own body appearance [2]. Individuals with AN-R may present with severe disturbances in body image that can potentially reach delusional proportions, and usually express extreme dissatisfaction with their appearance [3]. Body image disturbances do not only motivate severe dietary restriction and weight loss behaviours but also play a central role in the initiation, persistence, and relapse of AN-R [4]. From a phenomenological perspective, body image disturbances have been tightly linked in AN-R to an attitude of “objectification” toward the body, which may ultimately result in feeling extraneous from one’s own body and defining oneself only through objective measures [5]. Embodied cognition theories clarify that bodily representation does not only provide a schema to map external space but also play a major role in shaping our own basic sense of self and subjective experience [6–8]. Whereas disturbances in a broader notion of the concept of the Self have been discussed regarding AN-R [9], the relationship between eating disorders (EDs) and disturbances of the basic self appears to be unaddressed in the literature. This gap prompted our interest to empirically investigate different dimensions of self-experience and subjectivity in the domain of EDs and their relationship with body image, starting from AN-R. The issue is worth pursuing, in the light of both the clinical predictive value of body image distortion in AN-R and the lack of interventions successfully tackling this psychopathological feature [5].

The notion of anomalous self-experience (ASE) was originally formulated on the basis of accounts from patients with schizophrenia-spectrum disorders and represents trait-like changes at the basic level of self-awareness, including a pervasively diminished or unstable sense of self-awareness as well as abnormalities in bodily experience [10]. Starting from a classical phenomenological perspective, including Huber’s notion of basic symptoms, Parnas et al. have developed the examination of anomalous self-experience (EASE) scale as a semi-structured interview to guide such clinical exploration of ASEs [11].

This is the first study that capitalizing on an accurately selected group of individuals with AN-R at onset explored the impact of ASEs on abnormal body image attitude and ED symptomatology. We hypothesized that subjects with AN-R would report higher level of ASEs as compared to age- and educational level-matched healthy controls (HCs), and that there would be a relationship between ASEs and ED symptomatology. In addition, we sought to test whether abnormal body image attitudes mediated this relationship.

## Methods

### Sample and study design

This was a case–control analytic study. Sixty-seven treatment‐seeking female adolescents and young adults, aged from 14 to 21 (mean age 18.3 ± 2.4), with a diagnosis of AN-R at first-onset according to DSM-5 criteria were consecutively recruited from EDs specialized outpatient clinics of Agostino Gemelli University Hospital Foundation IRCCS—Catholic University of the Sacred Heart of Rome, Italy. To ensure inclusion of an accurately selected clinical group of individuals with first-onset AN-R, only patients admitted within a maximum of 1 year from the initiation of weight loss or insufficient age‐expected weight gain were considered eligible for the study. The Structured Clinical Interview for DSM-5 Clinician Version (SCID-5-CV [12]) was employed to establish AN-R diagnosis and psychiatric comorbidity. Furthermore, all patients were screened for Personality Disorders using the Structured Clinical Interview for DSM-5 Personality Disorders (SCID-5-PD [13]). Diagnostic interviews were conducted at study entrance by raters with extensive training and demonstrably high interrater reliability (*κ* > 0.8). Due to the focus on demanding EASE interviews, the following inclusion and exclusion criteria were strictly adopted to ensure a suitable study sample: all participants were to be in a stable medical phase of illness, euthymic according to psychometric evaluation (Hamilton Depression Rating Scale [14], HAM-D ≤ 7), and currently meet DSM-5 criteria for AN-R, without any past/current comorbid psychiatric disorder or substance use. Moreover, participants had to demonstrate adequate command over written and spoken Italian language and report cognitive function within the normal range according to Raven’s progressive matrices test [15]. Of the initial 67 clinical subjects, 40 participants with first-onset AN-R met study eligibility criteria and were included in the final clinical sample. Specifically, 20 individuals with AN-R were screened positive for current psychiatric comorbidities, and seven participants were excluded due to unstable medical conditions. Forty-five age and educational level-matched HCs were recruited through local online advertising. All HCs were screened for lifetime personal history of DSM‐IV‐TR Axis I and II disorders using the SCID‐I/NP [16] and SCID‐II [17] as well as for family history (up to second degree relatives) of mood disorders or schizophrenia. Participants with DSM‐IV‐TR Axis I or II disorders and/or family history of mood disorders or schizophrenia were excluded from the HC group. All other eligibility criteria were the same as those for the AN-R group. The final sample comprised 85 individuals, 40 with AN-R and 45 age, and educational level-matched HCs, a number that was comparable with previous EASE research assessing ASEs in psychiatric disorders outside the schizophrenia-spectrum [18–20].

The study was approved by the Agostino Gemelli University Hospital Foundation IRCCS—Catholic University of the Sacred Heart of Rome Ethics Committee and was undertaken in accordance with the Principles of Human Rights, as adopted by the World Medical Association at the 18th WMA General Assembly, Helsinki, Finland, June 1964, and subsequently amended at the 64th WMA General Assembly, Fortaleza, Brazil, October 2013. All participants gave their written informed consent to participate in the study after they had received a complete explanation of the procedures.

### Measures

#### Clinical parameters and anamnestic features

Age, weight, height, body mass index (BMI, calculated after measuring each participant’s weight and height), early weaning from breastfeeding, smoking status, demographics, family history of psychiatric disorders, duration of illness (the elapsed time between initiation of weight loss or insufficient age‐expected weight gain and study inclusion), and overall weight loss (the weight difference in kilograms between the beginning of the illness and the time of admission) were recorded for each patient at the time of admission.

#### Ease

Anomalous self-experience was explored through the EASE [11, 21], a checklist providing a framework for phenomenological-descriptive assessment of patients’ subjective experience. The EASE consists of the following main sections or domains: (1) disturbances of cognition and stream of consciousness, (2) disturbances of self-awareness and presence, (3) anomalous bodily experiences, (4) demarcation/transitivism, i.e., the ability to construct self-boundaries and to avoid undesired and passive permeation from outside, and (5) existential reorientation, i.e., reorientation of patient’s metaphysical worldview and/or value hierarchy/prioritisation, projects and interests, including solipsistic, centrality/self-reference, and magical ideas [11]. To ensure comparability with previous and ongoing studies on the EASE, Likert severity scores of EASE items were dichotomized by coding “not present” and “questionably present” as “not present” and “mild”, “moderate”, and “severe” as “present”. The participants were interviewed in a single session and all EASE interviews were audio and/or videotaped for transcription. In accordance with the conceptual principles of the scale, the interviews were conducted in a semi-structured fashion and a mutually interactive exploration was further encouraged throughout. In previous studies, the EASE has demonstrated to have high internal consistency as well as good interrater reliability. All interviews were conducted by the first authors (LM and EC), two board-certified psychiatrists with substantial clinical experience and extensive psychopathological background, as well as formal certification for using the EASE. To ensure assessment reproducibility, interrater reliability of the EASE ratings was regularly tested, with Kappa values of interviews transcripts comparable with those obtained in previous EASE studies (*κ* > 0.8) [20, 22, 23].

#### Body uneasiness test

Abnormal body image attitudes were assessed by the body uneasiness test (BUT [24, 25]), a 71-item self-report questionnaire that consists of two parts: BUT-A which targets a number of body image-related constructs, including weight phobia, body image concerns, avoidance, compulsive self-monitoring, detachment, and estrangement feelings toward one’s own body; and BUT-B, which looks at specific worries about particular body parts or functions. In keeping with previous validation studies, BUT-A scores were combined in a global severity index (GSI), whereas BUT-B scores were combined in two global measures (positive symptom total, PST, and positive symptom distress index, PSDI). Severity ratings of BUT items are expressed on a six-point Likert scale where zero indicating no problems in that specific item and 5 maximum severity. Higher scores indicate greater body uneasiness. The BUT proved established validity and reliability in several studies conducted on individuals with AN-R [26, 27].

#### Eating disorder examination questionnaire

Eating disorder symptoms severity was assessed with the eating disorder examination questionnaire (EDE-Q) version 6.0 [28, 29]. The EDE-Q is a 28-item self-report questionnaire measuring attitudinal features of EDs and core ED behaviours, such as eating, weight, and shape concerns, as well as dietary restraint during the past 28 days. Responses are rated on a 7-point Likert scale, with possible scores ranging from 0 to 6. EDE-Q yields four subscales that are combined in a global severity measure. Higher scores reflect greater severity of ED. The EDE-Q showed excellent reliability and validity in clinical samples involving individuals with AN-R [30].

### Statistical analyses

We compared individuals with AN-R and HC on demographic and clinical characteristics on the basis of contingency table/*χ*2 for categorical measures and Student’s *T* Test for continuous variables. The level of significance was of 5%.

### Distribution patterns of ASEs, body uneasiness, and ED symptom severity

For the aim of this study, we focused on the distribution patterns of ASEs, body uneasiness, and ED symptoms severity in patients with AN-R compared to HC. Therefore, we conducted a multivariate analysis of variance (MANOVA) using EASE total score, BUT-GSI, BUT-PST, BUT-PSDI, and EDE-Q as dependent variables and diagnosis as independent factor. When the initial model was significant, we conducted a series of one-way analyses of variance (ANOVA) to test differences between groups on dependent variables. We used a statistical model corrected for multiple comparisons according to the Bonferroni procedure (*p* < 0.05/number of comparisons) to minimise the likelihood of type I statistical errors. We examined possible multicollinearity between variables of interest using variance inflation factor (VIF) indicator obtained from a linear regression analysis.

### Mediation analysis

To test whether the relationship between ASEs and ED symptomatology in AN-R is mediated by abnormal body image attitude, we performed a mediation analysis in the clinical group using the PROCESS macro for SPSS [31]. Mediation implies a situation where the effect of the independent variable on the dependent variable can best be explained using mediator variables that are caused by the independent variable and are themselves a cause for the dependent variable. This means that instead of X (EASE total score) causing Y (EDE-Q) directly, X is causing the mediators M1 (BUT-GSI), M2 (BUT-PSDI), and M3 (BUT-PST), which in turn are causing Y (Model 6 in PROCESS). The relationships between the independent variable, the mediators, and the dependent variable are depicted in the form of a path diagram/model. For each path, the regression coefficients (betas) indicating the direction and magnitude of the effect of one variable on the other are shown. In the model, BMI was used as covariate, to rule out the potential confounding effect of the severity of medical condition. Mediation analyses were tested using a very conservative bootstrapping resampling procedure with bias-corrected confidence estimates. Bootstrapping is a strongly recommended process that treats the original sample as the population and resamples (with replacement) observations from within that sample thousands of times over to generate sample-based estimates of indirect effects and their standard errors [32]. The 95% confidence interval of the direct and indirect effects was obtained with 10 000 bootstrap samples. Direct and indirect effects are significant if confidence intervals do not contain a zero value. A *p* value < 0.01 has been defined as significant. All statistical analyses were performed using SPSS v. 25 (IBM Corp., Armonk, New York, USA).

## Results

As expected from the matching procedure, participants with AN-R and HCs did not significantly differ for age or educational level (Table 1). Patients with AN-R compared to HC reported significantly higher rates of early weaning from breastfeeding and lifetime psychotherapy. Patients also presented with significantly lower BMI with respect to HC (Table 1).

**Table 1:** Clinical and demographic characteristics of the sample

Characteristics	AN-R (*N* = 40)	HCs (*N* = 45)	Overall	*df*	*χ*^2^ or *F*	*p*
Age (*M* ± SD)	18.3 ± 2.3	18.2 ± 2.6	18.2 ± 2.5	1	0.1	892
Body mass index (*M* ± SD)	15.2 ± 1.3	21.1 ± 2.1	18.3 ± 5.4	1	232.7	< .001***
Years of education (*M* ± SD)	12 ± 2.3	12.8 ± 2.2	12.4 ± 2.3	1	2.5	.113
Overall weight loss (*M* ± SD)	9.2 ± 2.3					
Duration of illness in months (*M* ± SD)	6.2 ± 2.5					
Living alone (*n*%)	3 (7.5)	4 (8.9)	7 (8.2)	1	0.1	.816
Single child (*n*%)	9 (22.5)	4 (8.9)	13 (15.3)	1	3.1	.082
Early weaning from breastfeeding (*n*%)	5(12.5)	0 (0.0)	5 (5.9)	1	5.9	.014*
Occupation (*n*%)				1	3.4	.061
Employed	37 (92.5)	45 (100)	82 (96.5)			
Unemployed	3 (7.5)	0 (0.0)	3 (3.5)			
Smoking (*n*%)	10 (25.0)	13 (28.9)	23 (27.1)	1	0.2	.687
Family history of psychiatric disorders (*n*%)	9 (22.5)					
Receiving psychotherapy (*n*%)	32 (80.0)	14 (31.1)	46 (54.1)	1	20.3	< .001***

*AN-R* anorexia nervosa-restrictive subtype, *df* degrees of freedom, *F* value of variance of the group means, *HCs* healthy controls, *M* mean, *p* statistical significance, *SD* standard deviation, *χ*^*2*^ Chi-squared test

*(*p* < 0.05)

***(*p* < 0.001) significant results (in bold characters)

### Distribution patterns of ASEs, body uneasiness, and ED symptoms severity

The MANOVA indicated a significant global effect (Wilks' Lambda = 0.21, *F* = 56.20, *df* = 5, *p* < 0.001) of variables of interest on the two diagnostic groups. In particular, a series of univariate ANOVAs clarified that patients with AN-R significantly reported more ASEs experiences, more severe ED symptoms, and scored higher on BUT-GSI and BUT-PSDI as compared to HC (Table 2). There was no significance of multicollinearity in the model, as indicated by the fact that VIF of all variables of interest was < 4 [33]. EASE domains scores, as well as BUT-A and EDE-Q subscales, are provided in in Supplementary Information in Table S1, Table S2, and Table S3, respectively.

**Table 2:** Differences in EDE-Q, BUT, and EASE scores between the anorexia nervosa-restrictive type and healthy control samples

	AN-R (*N* = 40)	HCs (*N* = 45)	Overall	*df*	*F*	*ηp*^2^	*p*
EDE-Q (*M* ± SD)	3.3 ± 1.5	0.4 ± 0.4	1.7 ± 1.8	1	149.3	0.64	< 0.001***
BUT-GSI (*M* ± SD)	2.7 ± 1.2	0.7 ± 0.5	1.6 ± 1.3	1	86.7	0.51	< 0.001***
BUT-PST (*M* ± SD)	17.2 ± 8.1	15.9 ± 13.9	16.5 ± 11.4	1	0.2	0.01	0.606
BUT-PSDI (*M* ± SD)	2.8 ± 0.7	1.6 ± 0.4	2.1 ± 0.8	1	84.3	0.51	< 0.001***
EASE (*M* ± SD)	12.1 ± 4.5	1.0 ± 1.0	6.2 ± 6.4	1	255.3	0.75	< 0.001***

Significant results for *p* < 0.001 (in bold characters)

*AN-R* anorexia nervosa-restrictive subtype, *BUT-GSI* body uneasiness test—global severity index, *BUT-PSDI* body uneasiness test-positive symptoms distress index, *BUT-PST* body uneasiness test-positive symptoms total, *df* degrees of freedom, *EASE* examination of anomalous self-experience scale, *EDE-Q* eating disorder examination questionnaire, *F* value of variance of the group means, *HCs* healthy controls, *M* mean, *p* statistical significance, *SD* standard deviation, *ηp*^*2*^ partial eta-squared measure of effect size

### Mediation analysis

**Fig. 1:**
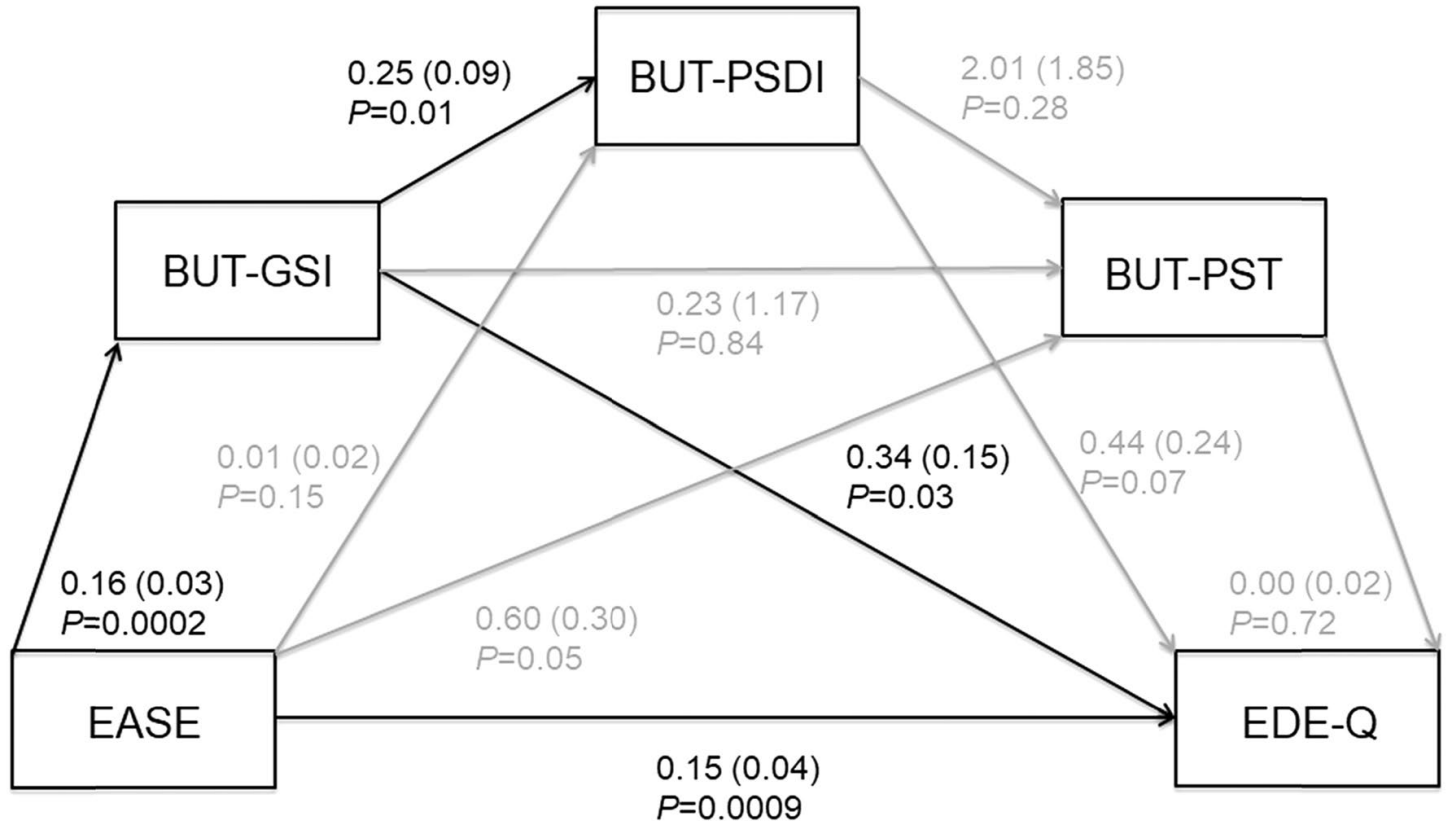
The direct and indirect effect of ASEs on ED Symptoms Severity. Path diagram of the mediation model (X = EASE through the examination of anomalous self-experience scale), Y = EDE-Q through the eating disorder examination questionnaire, M1 = BUT-GSI, M2 = BUT-PSDI, M3 = BUT-PST measured through the body uneasiness test. Each effect shows coefficients (standard errors) and *p* values. Black arrows represent significant effects; grey arrows represent not significant effects

In the group of patients with AN-R (*n* = 40), we found a direct effect of ASEs on ED symptom severity (*p* = 0.009; bootstrapped LLCI = 0.067, ULCI = 0.240). We also found that the relationship between ASEs and ED symptomatology was partly mediated by BUT-GSI. Under the bootstrapped 95% confidence interval, the significant indirect effect was 0.05 and did not contain 0 (LLCI = 0.001, ULCI = 0.172). Specifically, we found that EASE total score was positively related to BUT-GSI, which in turn was positively related to EDE-Q. BUT-GSI was significantly related to BUT-PSDI (Fig. 1). BMI was not significant in any of the effects tested (*p* > 0.05). No other significant indirect effects were observed in the model.

## Discussion

Consistent with our main hypothesis, there were significant differences in the level of ASEs between the two groups, with overall higher EASE total and domain scores in individuals with AN-R as compared to HCs. Furthermore, among those diagnosed with AN-R, we detected a significant direct effect between ASEs and ED symptomatology that, after controlling for the effect of body image, resulted partially mediated by abnormal body image attitude. To the very best of our knowledge, no previous studies have investigated this relationship in AN-R.

The relationship between AN and schizophrenia-spectrum disorders, including disturbances of the basic self, is empirically under-researched to date. During the mid-20th century, AN was described as one of numerous “pseudo-neurotic” manifestations among schizophrenia-spectrum [34–37]. In accordance with this conceptual framework, Hilde Bruch [38] proposed a descriptive and theoretical model defining AN as a self-disorder, where dietary restriction is conceived of as an effort to camouflage underlying deficits in the structure of the Self. Of note, a recent study assessing the distribution of self-disturbance in ultra-high risk and first-episode psychosis individuals found ASEs to predict a number of psychopathological dimensions, including ED symptoms [39].

Contemporary phenomenology and cognitive neuroscience make distinction between the “narrative” self and the “minimal” or “core/basic” Self [40, 41]. The notion of core self refers to the first-personal manifestation of all experience and involves a sense of self-coincidence, “me/not me” demarcation, as well as psychosomatic unity [31]. Patients with AN-R can describe such structural abnormalities of self-experience, including incomplete sense of body ownership, along with an ephemeral sense of self-presence and subjective feeling of “over-adaptation” [35, 42].

Although the exact pathways linking AN-R to self-disturbance remain to be identified, in the present study, we clarified that the impact of ASEs on ED symptomatology is direct, and partially mediated by alterations in body image. Specifically, we found that EASE total scores were positively related to BUT-GSI, which in turn were related to ED severity. Body image, as a multifaceted construct, entails the subject’s perceptual experience of her/his own body, the subject’s conceptual understanding of the body in general, as well as the subject’s emotional attitude toward her/his own body [43]. Of note, body image has been linked to the subjective experience of embodiment, i.e., the consciousness of one’s sense of self is localized within one’s bodily borders [44]. From a phenomenological perspective, one could speculate that body image may be adversely related in AN-R to a hyper-reflexive (i.e., objectified) experience of the bodily self, whereas ED symptoms may entail compensatory aspects attempting to reconnect with a sense of bodily presence, as well as enforcing identity when the basic self is unstable and challenged [39]. In other words, a disembodied attitude toward one’s own body may therefore characterise AN-R at level of minimal self-awareness (for a clinical description, please see the clinical vignette in Supplementary Information).

Before summarizing study conclusions, we must acknowledge some potential limitations that might limit the generalizability of our results. First, due to the lack of diagnostic blinding of the assessments, the study’s internal validity was potentially affected, yielding a risk of a systematic bias. However, as it has previously been argued, a focus on both a thorough lifetime social and clinical exploration is imperative to satisfy the basic tenets of a phenomenological interviewing approach, which is indispensable to faithfully explore disorders of subjectivity [20]. Second, to ensure the conceptual and methodological validity of the study, as well as optimal conditions for EASE interviews, a sample of subjects with AN-R in a stable phase of illness and without psychiatric comorbidity was selected. Accordingly, this issue might have led to a selection bias. However, selecting a well-demarcated clinical group of subjects with AN-R at onset can be also considered as study strength. Finally, the reliability of EDs symptoms severity as indexed by self-administered questionnaires may be partially biased, as there is evidence that individuals with AN-R may under-report dietary restraint [45].

### What is already known on this subject?

A struggle for identity along with an incomplete sense of selfhood, including poor coherence of body representations, is commonly experienced by individuals with AN-R. However, the relationship between ED symptomatology and disturbances of the basic self has not received adequate empirical testing yet.

### What does this study add?

This is the first study to explore the presence of ASEs in an accurately selected group of patients with AN-R at onset, and to clarify the impact of ASEs on both body image disturbance and ED symptoms. Accordingly, based on our findings, a broader exploration of possible transdiagnostic features in AN, including explorations of different dimensions of self-experience and intersubjectivity, may contribute to shed further light on the clinical phenomenology of the disorder, and to tailor therapeutic interventions.

## Supplementary Information

Below is the link to the electronic supplementary material.

Supplementary file1 (DOCX 24 KB)

## Data Availability

The data that support the findings of this study are available upon reasonable request from the corresponding author. The data are not publicly available due to privacy and/or ethical restrictions.
