# Supplementary material for: Anomalous self-experience, body image disturbance, and eating disorder symptomatology in first-onset anorexia nervosa
Source: Eat Weight Disord. 2021 Mar 4;27(1):101–8. doi: 10.1007/s40519-021-01145-0 (PMC8860951; doi:10.1007/s40519-021-01145-0)
Supplement: Supplementary file 1 — Supplementary file1 (DOCX 24 KB) [file 40519_2021_1145_MOESM1_ESM.docx]

| EASE | AN  *(N=40)* | HC  *(N=45)* | Overall |
| --- | --- | --- | --- |
| *Domain I* | 4.3 ± 1.8 | 0.4 ± 0.6 | 2.2 ± 2.3 |
| *Domain II* | 5.7 ± 2.3 | 0.6 ± 0.8 | 3.0 ± 3.1 |
| *Domain III* | 1.7 ± 1.1 | 0.0 ± 0.1 | 0.8 ± 1.1 |
| *Domain IV* | 0.2 ± 0.5 | 0.0 ± 0.0 | 0.1 ± 0.4 |
| *Domain V* | 0.1 ± 0.2 | 0.0 ± 0.0 | 0.0 ± 0.1 |

**Table S1.** EASE domains scores of AN-R and HC groups.

Abbreviations: AN-R = Anorexia Nervosa-restrictive subtype; EASE = Examination of Anomalous Self-Experience scale; M =mean; SD = standard deviation.

**Table S2.** BUT-A subscales scores of AN-R and HC groups.

| BUT-A | AN  *(N=40)* | HC  *(N=45)* | Overall |
| --- | --- | --- | --- |
| *Weight Phobia* | 3.0 ± 1.4 | 1.2 ± 1.0 | 2.1 ± 2.5 |
| *Body Image Concern* | 3.1 ± 1.2 | 0.5 ± 0.5 | 1.7 ± 1.6 |
| *Avoidance* | 2.0 ± 1.5 | 0.1 ± 0.3 | 1.0 ± 1.4 |
| *Compulsive Self-Monitoring* | 2.3 ± 1.4 | 0.3 ± 0.4 | 1.2 ± 1.4 |
| *Depersonalization* | 2.5 ± 1.4 | 0.2 ± 0.3 | 1.3 ± 1.5 |

Abbreviations: AN-R = Anorexia Nervosa-restrictive subtype; HC = healthy controls; BUT-A = Body Uneasiness Test – part A; M = mean; SD = standard deviation.

**Table S3.** EDE-Q subscales scores of AN-R and HC groups.

| EDE-Q | AN  *(N=40)* | HC  *(N=45)* | Overall |
| --- | --- | --- | --- |
| *Restraint* | 2.9 ± 1.9 | 0.4 ± 0.6 | 1.6 ± 1.8 |
| *Eating Concern* | 2.7 ± 1.3 | 0.2 ± 0.4 | 1.4 ± 1.6 |
| *Shape Concern* | 4.0 ± 1.6 | 0.6 ± 0.8 | 2.2 ± 2.1 |
| *Weight Concern* | 3.7 ± 1.6 | 0.5 ± 0.7 | 2.0 ± 2.0 |

Abbreviations: AN-R = Anorexia Nervosa-restrictive subtype; HC = healthy controls; EDE-Q = Eating Disorder Examination Questionnaire; M = mean; SD = standard deviation.

*Clinical Vignette*

Sara is an eighteen-year-old. She was brought to our outpatient eating disorder treatment service by her mother, turned desperate for her daughter’s fasting behaviour and consequent huge weight loss. She attends the senior year of high school and she was a straight-A student until a few months ago; She has been a ballet dancer since she was 5, but recently she dropped the class. Before symptoms onset, she was particularly close to her mother with whom she used to share everything. She now turned oppositional and lost any vital impetus towards things. Before starting EASE interview, Sara was asked how the food restriction began. “At a certain point of my life I realized that everything started to get out of my hands: the time, the fiancée along with my friends and interests. I did not recognize myself anymore. I started to question whether my boyfriend was the one I have loved or not. To be honest, I think I got engaged with him because I had to: I could not be the only one without. What would my friends think of me? I did not know what either my passions or my aptitudes were. It seemed like everyone at this step of (the) life had a dream to fulfil. What about me? I was way over my head, and I focused on food. I started with a vegan kick to imitate my best friend. She was full of ideals and I thought that sharing with her this battle for animal safeguard could have been useful for me too: eventually I had a role in the world. As I was losing weight, I realized my actions were effective, for once. Finally, I had something under my control in this ocean of uncertainties”. Suddenly, we were in the core part of the interview. She showed a huge indecision in her choices, even for the easiest ones: just the chance to switch on or off the light of the visit room was paralyzing her. She asked me to take such a decision for her. She is often not able to choose what is right or not, delegating to others the final judgment. In these occasions a sense of bewilderment imbues her. She cannot define herself and so she cannot define her will, her desires. She feels like she misses inner standpoint. She does not know who she is. This pervasive lack of identity results in a great sense of inner emptiness, to the point that she often needs to look in the mirror to know if she exists. She often wonders about everything, about the meaning of the most banal things, those that are obvious for everyone else. She also wonders about her stream of consciousness as she speaks with me; this is causing her a feeling of strangeness to what is happening, as if the inner reality were detaching itself from the flow of outer events. Towards the world given as an external reality, she feels muffled especially when she sits at the table and approaches food, feeling like being in a bubble she cannot get out of and in which contact with others is impossible. She feels awfully distant from other people. The society we live in, she states, is short-sighted because it judges everyone through their body, a body that is worth little more than an object. It is a sort of shell, a sarcophagus, which usually does not correspond to what we are inside. I ask her how she would represent herself if she had the chance. She replies she does not know. She does not feel “neither fish nor fowl”. That body is stranger, alien. She feels like her body does not really fit in with her. She burst into tears: she has a marked ease in crying when we explore her body image. She does not want to gain weight; she would lose everything. She started to feel someone right when she started her vegan diet. The friend who inspired her was a very close one, to the point that when they spent a lot of time together, she seemed to assume her movements, her accent. Sometimes it felt like she was her and that scared her a little. She was confused. The way to differentiate herself was immediately offered by the weight loss: her personal battle was no longer animals, but her body control. That body, felt so distant, had become the instrument to exercise its power in the world. It was a way of feeling an intentional being with its own effectiveness.
